# Detection of Acromion Types in Shoulder Magnetic Resonance Image Examination with Developed Convolutional Neural Network and Textural-Based Content-Based Image Retrieval System

**DOI:** 10.3390/jcm14020505

**Published:** 2025-01-14

**Authors:** Mehmet Akçiçek, Mücahit Karaduman, Bülent Petik, Serkan Ünlü, Hursit Burak Mutlu, Muhammed Yildirim

**Affiliations:** 1 Department of Radiology, Faculty of Medicine, Malatya Turgut Ozal University, 44210 Malatya, Turkey; bulent.petik@ozal.edu.tr; 2 Department of Software Engineering, Malatya Turgut Ozal University, 44210 Malatya, Turkey; mucahit.karaduman@ozal.edu.tr; 3 Department of Radiology, Malatya Training and Research Hospital, 44330 Malatya, Turkey; serkanunlu19@yahoo.com; 4 Department of Computer Engineering, Malatya Turgut Ozal University, 44210 Malatya, Turkey; burakmutlu44@gmail.com (H.B.M.); muhammed.yildirim@ozal.edu.tr (M.Y.)

**Keywords:** CBIR, CNN, feature selection, retrieval, shoulder acromion

## Abstract

**Background:** The morphological type of the acromion may play a role in the etiopathogenesis of various pathologies, such as shoulder impingement syndrome and rotator cuff disorders. Therefore, it is important to determine the acromion’s morphological types accurately and quickly. In this study, it was aimed to detect the acromion shape, which is one of the etiological causes of chronic shoulder disorders that may cause a decrease in work capacity and quality of life, on shoulder MR images by developing a new model for image retrieval in Content-Based Image Retrieval (CBIR) systems. **Methods:** Image retrieval was performed in CBIR systems using Convolutional Neural Network (CNN) architectures and textural-based methods as the basis. Feature maps of the images were extracted to measure image similarities in the developed CBIR system. For feature map extraction, feature extraction was performed with Histogram of Gradient (HOG), Local Binary Pattern (LBP), Darknet53, and Densenet201 architectures, and the Minimum Redundancy Maximum Relevance (mRMR) feature selection method was used for feature selection. The feature maps obtained after the dimensionality reduction process were combined. The Euclidean distance and Peak Signal-to-Noise Ratio (PSNR) were used as similarity measurement methods. Image retrieval was performed using features obtained from CNN architectures and textural-based models to compare the performance of the proposed method. **Results:** The highest Average Precision (AP) value was reached in the PSNR similarity measurement method with 0.76 in the proposed model. **Conclusions:** The proposed model is promising for accurately and rapidly determining morphological types of the acromion, thus aiding in the diagnosis and understanding of chronic shoulder disorders.

## 1. Introduction

The acromion, which articulates with the clavicle, is formed as a posterolateral extension of the scapular spine over the glenoid and is the origin of the deltoid and trapezius muscles [1]. The morphological type of the acromion may play a role in the etiopathogenesis of various pathologies, such as shoulder impingement syndrome and rotator cuff disorders [2]. Rotator cuff disorders and impingement syndrome are the most common causes of chronic shoulder pain and disability. Among the rotator cuff, which consists of four muscles and maintains the stability and strength of the shoulder joint, the supraspinatus tendon is the most frequently injured one [3]. Risk factors grouped as internal, such as hypovascular and collagen fiber anomalies, or external, such as subacromial impingement and acromion morphology, have been stated in the etiopathogenesis of a rotator cuff tear (RT) [4]. When the literature is reviewed, the etiopathogenesis of RCT remains controversial. The acromion part of the scapula and its morphology may play a role in various shoulder diseases such as RCT [2]. Bigliani et al. were the first to discuss the clinical significance of acromial morphology, stressing the significance of an anterior acromial down slope in terms of RCT [5]. Bigliani classification seems to be the first classification system that tried to define acromial morphology. However, some angles are used to define acromial morphology on lateral radiographs; acromial angle classification on direct radiography divides the acromion into three types, and on MRI, the acromion is divided into three types by evaluating its lower surface and length [6].

Currently, acromial shapes are generally divided into four types: type I (flat), type II (curved), type III (hooked), and type IV (convex) [7,8]. Hooked-type and curved-type acromions have been found to be strongly associated with RCT and may contribute to tendon traction injury, whereas flat-type acromions may contribute to rotator cuff impingement insignificantly [5]. According to Paraskevas et al., the hooked acromion’s smaller subacromial area may result in more frequent rotator cuff tendon impingement and eventual tearing [9]. In the study by Mohamed et al., a significant correlation was noted between a Type 3 acromion and RCT [10]. Some studies also reported that the RCT size was significantly larger in type III [4,11].

According to measures like the acromial type, acromial inclination, acromial slope, lateral acromial angle, and acromial index used in routine radiographs, the morphology of the acromion appears to be associated with the etiopathogenesis of RCT [7]. However, it is quite difficult to visualize and distinguish the shape of the acromion with a plain radiograph. Magnetic resonance imaging (MRI) allows the delineation of the acromion shape and provides a better assessment of the morphological factors of the acromion [12].

Since different imaging techniques and views may affect acromial classification, highly standardized methods should be aimed for in clinical practice. Thanks to developing technology and current feature extraction methods, CBIR systems have recently been frequently used in disease detection [13]. In the CBIR system developed in this article, feature maps used in the proposed model are extracted from the database and each queried image. Feature maps of the images queried in the proposed CBIR method and included in the data set are made using Euclidean distance and PSNR similarity measurement methods. In this calculation, the 20 images with the closest similarity are retrieved. Our article compared our proposed architectural model with Densenet201, Darknet53, Efficientnetb0, NasnetMobile, LBP, and HOG architectures in the CBIR system. In this study, Euclidean and PSNR similarity measurement methods were evaluated in the CBIR system [14].

We aimed to detect acromion shape types, which are among the etiological factors of chronic shoulder pain and disability, which may cause a decrease in work capacity and quality of life, with the CBIR-based system developed on shoulder MRI images. Thus, our aim was to take into account the acromial morphology, which may play a role in the etiopathogenesis, and in the evaluation of patients with shoulder pathologies such as shoulder impingement syndrome or RCT, and also to contribute to the studies to be carried out on this subject in the coming period.

## 2. Material and Methods

This section provides information about the MR data set consisting of shoulder acromion types used in our CBIR system, the feature selection and extraction methods, and the general structure of the CBIR method.

### 2.1. Data Set

This study was conducted after approval from Malatya Turgut Ozal University’s non-interventional clinical research ethics committee (Approval number: E-30785963-020-220615). Every procedure used in the research involving human subjects complied with the 1964 Helsinki Declaration, its subsequent amendments, the institutional and/or national research committee’s ethical requirements, or similar norms.

The Training and Research Hospital picture-archiving and communication system (PACS) was used to evaluate the shoulder MRI. Between 1 May 2023 and 01 May 2024, 3896 right and left shoulder MRI images of patients aged 18–60, taken for various reasons, were scanned. All the scans were performed on two 1.5-T magnets (MAGNETOM Amira, Siemens Healthcare, Erlangen, Germany) with a dedicated shoulder coil.

**Figure 1:**
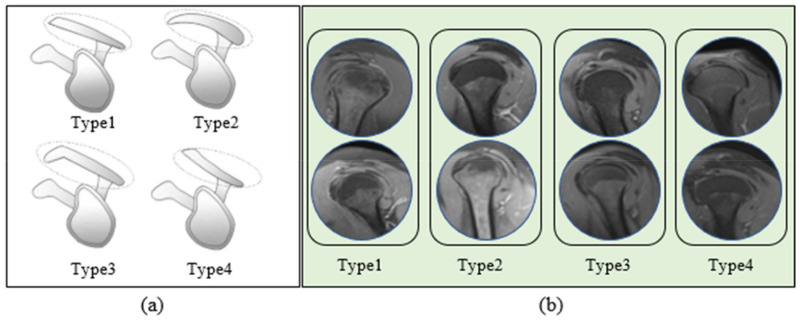
Graphical representation of acromion subtypes (**a**) and sample images of the shoulder MRI data set’s (**b**). Figure 1a presents a graphic representation of acromion subtypes . Moreover, Figure 1b presents (**a**) and sample images of the shoulder MRI data set’s Type 1, Type 2, Type 3, and Type 4 classes (**b**).

Bone tumors, post-traumatic arthritis, previous shoulder surgery, congenital deformities, unstable shoulders, calcified tendinitis, and artifactual images were exclusion criteria. Images were reviewed by three radiologists with at least ten years of experience. The study includes images of 748 patients who fit the requirements. PD TSE fat sat sagittal images were used when typing the shoulder acromion. Acromial shapes are divided into four types: type I (flat) (178 images), type II (curved) (337 images), type III (hooked) (104 images), and type IV (convex) (129 images). A graphical representation of acromion subtypes and sample images from the data set are given in Figure 1.

### 2.2. Feature Extraction, Feature Selection, CBIR, and Performance Measurement Metrics

The deep learning method combines several processing layers and abstraction structures to learn data representations [15]. Choosing the appropriate features is crucial to the effectiveness of machine learning techniques [16]. Various preprocessing, dimensionality reduction, feature selection, etc., reveal these features. In computer vision and image processing, HOG is an effective feature descriptor for object recognition [17]. The LBP operator labels each pixel in an image by thresholding its neighboring pixels in the surrounding 3 × 3 neighborhood according to the center pixel value. The surrounding pixel is assigned the value 1 if its value is larger than or equal to the center pixel and 0 otherwise. For a neighborhood, an 8-bit LBP code is thus generated. The mRMR algorithm’s filtering technique seeks to minimize redundancy among the features it selects while choosing the most pertinent characteristics with class labels [18].

The purpose of the CBIR system is to display the images in the data set that show the closest similarity to the queried image. CBIR systems are computer vision applications developed to solve the problem of retrieving the desired image from an extensive database of digital images [19]. CBIR consists of two primary stages: feature extraction and similarity calculation. The feature extraction phase is first performed in the image database. In the relevant process, a feature database is created with color, texture, and shape descriptors. In the similarity calculation phase, the feature extracted from the query image is compared with those in the feature database. In CBIR, the query is not performed at the pixel level but at the level of equal-length vectors representing the image. The image representation power, number, and computational complexity of the resulting vectors affect the methods’ performance. CBIR’s other elements are the user feedback, image database, and performance evaluation [20].

Distance or similarity measurement methods are used to determine the similarity of images for which feature maps are extracted. Mean Squared Error (MSE), Peak Signal–Noise Ratio (PSNR), and Structural Similarity Index (SSIM) measurement metrics are generally used as similarity measurement methods. As for distance measurement methods, Euclidean, quadratic, cosine, and histogram intersection measurements are among the frequently used distance measurement metrics [21,22]. Recall rate and precision factors are frequently used to calculate an information retrieval system’s effectiveness. The recall measure is the extent to which the search results are comprehensive. This is accomplished by comparing the quantity of “found, relevant” images with “not found, relevant images.” Stated differently, recall represents the probability of discovering a pertinent image. The precision of a search result is determined by dividing the total number of relevant images found by the total number of irrelevant images found. Sensitivity is a measure of an image’s likelihood of being relevant. Essentially, both values fall between 0 and 1. In this case, 1 would be the ideal value [23,24,25].

### 2.3. CBIR-Based System Proposed for Detection of Acromion Types

**Figure 2:**
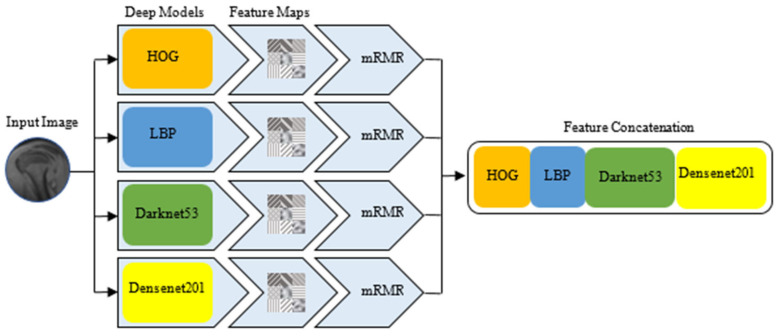
Extraction of feature map for CBIR method.

The first step of the CBIR-based model developed in this study is feature extraction. CNN-based Darknet53, Densenet201, textural-based HOG, and LBP architectures were used in the model to be used in the developed CBIR system. Feature maps of the images were extracted separately using each relevant method. To remove non-distinctive features in the feature vectors of the four models whose feature maps were drawn and to increase the working performance, feature vector dimension reduction was performed with the mRMR optimization method. In the feature vector size reduction performed with mRMR, the HOG feature vector size was optimized as 400, the LBP feature vector size was optimized as 500, the Darknet53 feature vector size was optimized as 400, and the Densenet201 feature vector size was optimized as 400. Then, the feature maps optimized by the mRMR dimensionality reduction method were combined. In this case, a 748 × 1700 feature map of 748 images in the database was obtained. The flow diagram of the proposed block for feature map extraction to be used in the CBIR system is presented in Figure 2.

**Figure 3:**
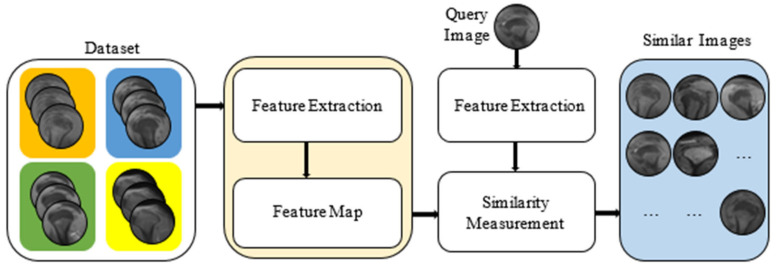
The proposed model in the CBIR system.

For the CBIR model, built to determine the types of shoulder omicrons, a feature map of the image to be searched was created in both the recommended and alternative models. The PSNR similarity and Euclidean distance measuring methods were used to compare the models. An interpolated 11-point sequential reach curve was used to measure the P-R curve to assess the comparability. The block diagram of our CBIR-based model, which was developed by including similarity measurement metrics, is given in Figure 3.

In the developed model, feature extraction is performed for training and testing data. Then, using similarity measurement metrics, similar images of an image are drawn from the data set.

## 3. Results

This section emphasizes the results obtained in determining shoulder acromion types. The MATLAB 2023b environment was used to capture the application results on a PC running Windows 10 64-bit, with an Intel i7 processor and 16 GB of RAM.

In the paper, the suggested approach was compared with the models of Densenet201, Darknet53, Efficientnetb0, NasnetMobile, LBP, and HOG. The Euclidean distance measurement and PSNR similarity methods were used to calculate the similarity between the images and compare the feature maps extracted from this architecture. By determining the precision and sensitivity values for each retrieved image by the sequential retrieval of the listed images, an interpolated 11-point sequential retrieval P-R curve was produced for this comparison. The Average Precision (AP) values derived from the P-R curve were compared to the designs.

**Figure 4:**
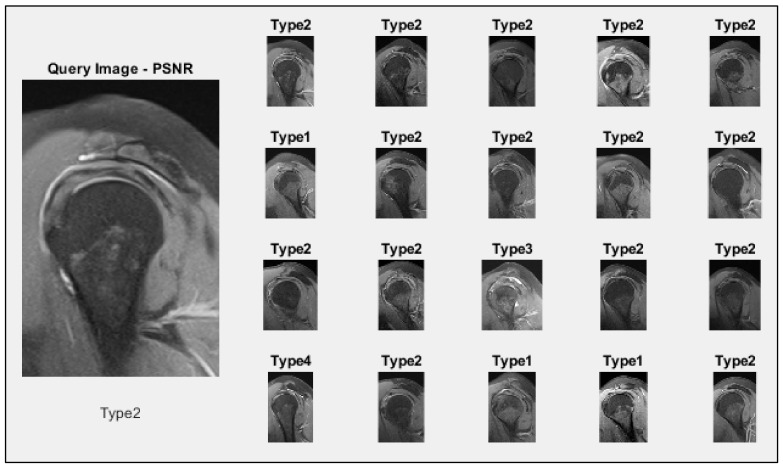
Twenty images retrieved for a queried image.

As an illustration, the suggested CBIR system can be presented roughly; as seen in Figure 4, 20 similar images were retrieved in the query image of the Type 2 class given for the query from the data set. As a result of the query, 20 images were retrieved, 15 of which belonged to Type 2, 3 to Type 1, 1 to Type 3, and 1 to Type 4 classes.

This paper used the P-R curve and the AP value to assess interpolated 11-point sequential retrieval. The average P-R curve was obtained by individually querying the images in four classes. Twenty images were obtained from the CBIR system after individual queries were made for each type of image. The average P-R curve was created by evaluating each class’s queried and retrieved images.

**Figure 5:**
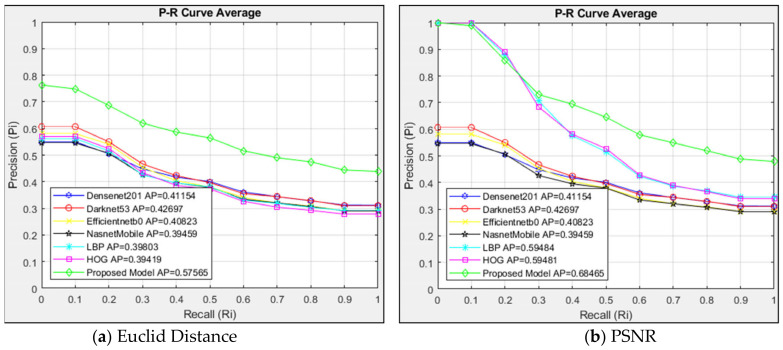
Average P-R curve of Type 1 class.

The average P-R curve of the twenty images returned in each query was used to analyze the 748 images in the data set after the P-R curves of the four classes had been assessed independently. The architectures were also evaluated using the Euclidean distance measurement method in Figure 5a and the PSNR similarity measurement method in Figure 5b. Twenty related images were found in the data set after the CBIR system searched 178 images in the Type 1 class.

In Figure 5a, models are evaluated with Euclidean distance. In the models compared with Euclidean distance measurement, the proposed model showed higher success than other architectures with AP = 0.58. The proposed model showed higher resistance than other architectures in the case of R = 0. The six architectures compared showed close performance. The proposed model maintained its success with a clear difference compared to the six compared architectures.

The models are compared using the PSNR similarity measuring method in Figure 5b. With the PSNR method, the proposed model showed high success with AP = 0.69 in images in the Type 1 class. Although HOG and LBP architectures were more successful than the proposed model up to R = 0.2, they showed less resistance than the proposed model after R = 0.3. Comparing Figure 5a,b, it can be shown that, in the suggested approach, the PSNR technique performed better than the Euclidean method in extracting comparable images with an AP = 0.69. Despite the Euclidean method’s lesser sensitivity, the PSNR approach produced a higher sensitivity at R = 0. While the HOG and LBP architectures could not demonstrate a discernible sensitivity in the Euclidean technique up to R = 0.2, the PSNR method produced results nearly identical to the developed model.

The proposed model was successful in both methods, compared with the interpolated 11-point sequential retrieval P-R curve in the Type 1 class. The Euclidean method had AP = 0.58, and the PSNR method had AP = 0.69. When the Euclidean and PSNR methods are compared, the PSNR method shows higher sensitivity in image retrieval than the Euclidean method, with AP = 0.69.

**Figure 6:**
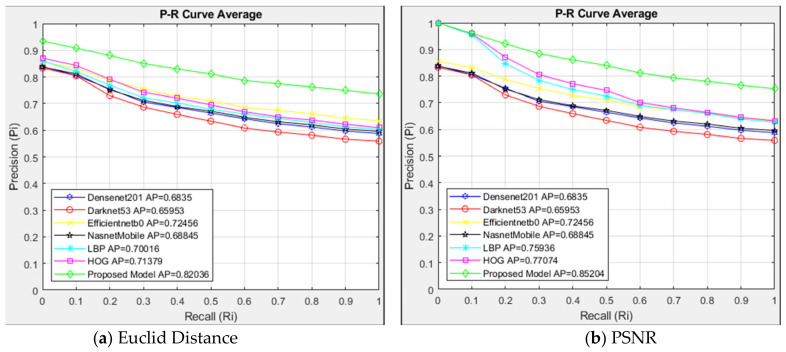
Average P-R curve of Type 2 class.

The suggested model and alternative architectures are contrasted with the interpolated 11-point sequential retrieval P-R curve of Type 2 class images in Figure 6. For every 337 images in the Type 2 class that the CBIR system searched, 20 identical images were returned, resulting in a list of 6740 images.

In Figure 6a, models are evaluated with Euclidean distance. In the models compared with Euclidean distance measurement, the proposed model showed higher success than other architectures with AP = 0.82. The proposed model showed higher resistance than other architectures in R = 0. The six architectures compared showed close performance. The Darknet53 architecture showed the lowest sensitivity with AP = 0.66. The models are compared using the PSNR similarity measuring method in Figure 6b. The suggested model demonstrated excellent performance with AP = 0.85 in Type 2 class images using the PSNR approach. Although HOG and LBP architectures had the same success as the proposed model up to R = 0.1, they showed less resistance than the proposed model after R = 0.2. Comparing Figure 6a,b, the PSNR approach outperformed the Euclidean method in the suggested model for identical image retrieval, with an AP = 0.85.

**Figure 7:**
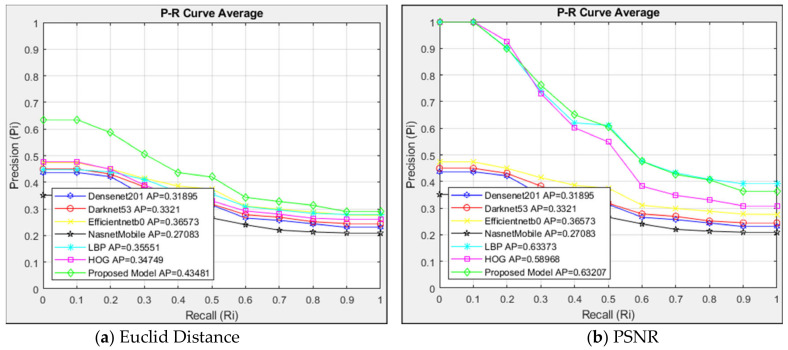
Average P-R curve of Type 3 class.

The suggested model and alternative architectures are contrasted with the interpolated 11-point sequential retrieval P-R curve of Type 3 class images in Figure 7. The architectures were also evaluated using the Euclidean distance measurement method in Figure 7a and the PSNR similarity measurement method in Figure 7b. The CBIR system queried 104 images in the Type 3 class, and 20 similar images in the data set were retrieved. Since 20 similar images are retrieved for each image queried in the Type 3 class, 2080 images are listed.

Euclidean distance measurement is used to evaluate the structures in Figure 7a. The proposed model outperformed other designs with an AP = 0.44 in the architectures compared with Euclidean distance measurement. When R = 0, the proposed architecture had more resistance at first than other architectures. The proposed model had a sensitivity comparable to Efficientnetb0 and LBP architectures when R = 0.6 was used. The proposed approach performed well up to R = 0.6, but its sensitivity was comparable to alternative architectures after that point. With an AP value of 0.27, the NasnetMobile architecture exhibited the most minor sensitivity. Figure 7b compares the models with the PSNR similarity measurement. The PSNR method showed high success with the proposed model, and LBP AP = 0.63 for images in the Type 3 class. Although HOG and LBP models were close to the proposed model up to R = 0.4, HOG showed less resistance after R = 0.4.

The suggested model achieved success with AP = 0.44 in the Euclidean approach when compared to the interpolated 11-point sequential retrieval P-R curve in the Type 3 class. In the PSNR method, the proposed model and LBP succeeded in both methods, with AP = 0.63. When the Euclidean and PSNR methods were compared, the PSNR method showed higher sensitivity in image retrieval than the Euclidean method with AP = 0.63.

**Figure 8:**
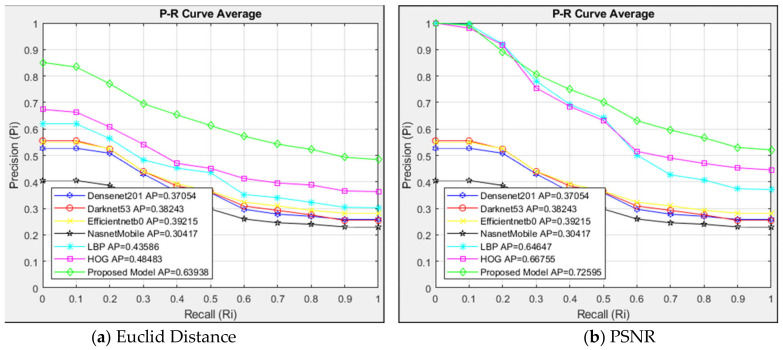
Average P-R curve of Type 4.

Out of the 129 images of the Type 4 class that the CBIR system queried, 2580 images are listed because each query yields 20 similar images. The suggested model and alternative architectures are contrasted with the interpolated 11-point sequential retrieval P-R curve of Type 4 class images in Figure 8.

The structures in Figure 8a are evaluated using Euclidean distance measurement. The suggested model performed better than other structures with an AP = 0.64 with the Euclidean distance measurement compared to the architectures. In the case of R = 0, the suggested model had stronger resistance than other structures from the outset and higher sensitivity than the compared six architectures. With an AP value of 0.30, the NasnetMobile architecture exhibited the least sensitivity. Figure 8b compares the architectures using the PSNR similarity measuring method. The suggested model performed well with the PSNR approach, achieving an AP = 0.73 in the Type 4 class images. The HOG and LBP architectures exhibited less resistance after R = 0.2, although they were similar to the suggested model up to that point.

When Figure 8a,b are compared, the PSNR method was more successful in retrieving similar images with AP = 0.73 than the Euclidean method in the proposed model. Although the Euclidean method showed a lower sensitivity at R = 0, the PSNR method achieved a higher sensitivity. HOG and LBP architectures are more sensitive to the PSNR method than the Euclidean method. When the Euclidean and PSNR methods were compared, the PSNR method showed higher sensitivity in image retrieval than the Euclidean method with AP = 0.73.

**Figure 9:**
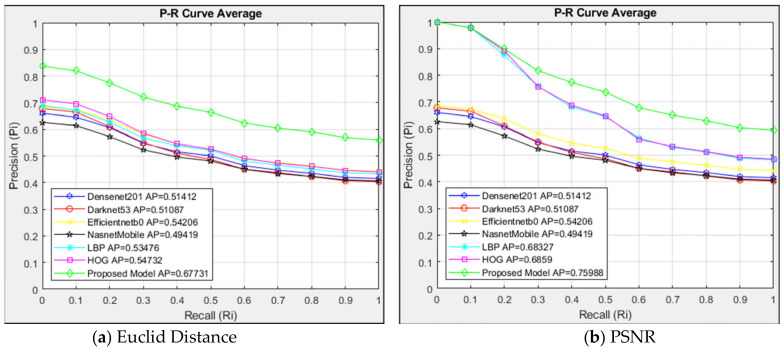
Average P-R curve of data set.

The proposed and alternative models are compared with the interpolated 11-point sequential retrieval P-R curve of every image in the data set in Figure 9. The architectures were also evaluated using the Euclidean distance measurement in Figure 9a and the PSNR similarity measurement method in Figure 9b. A total of 20 images similar to the 748 images in the data set queried in the CBIR system were obtained. A total of 14,960 images were listed since 20 comparable images were returned for every image queried in the data set.

The structures in Figure 9a are evaluated using Euclidean distance measurement. The suggested model performed better than other structures, with an AP = 0.68 when the architectures were compared using the Euclidean distance measurement. The architectures are compared using the PSNR similarity measuring method in Figure 9b. The suggested model performed very well with the PSNR approach, achieving an AP value of 0.76 in the data set’s images. The HOG and LBP architectures exhibited less resistance after R = 0.2, although they were similar to the suggested model up to that point.

Comparing Figure 9a,b, it can be seen that, in the suggested model, the PSNR technique outperformed the Euclidean method in similar image access, with an AP = 0.76. Despite the Euclidean method’s lesser sensitivity, the PSNR approach produced a higher sensitivity at R = 0. Compared to the Euclidean technique, the PSNR method is more sensitive to HOG and LBP architectures.

With AP = 0.68 in the Euclidean method and AP = 0.76 in the PSNR method, the suggested model performed well in both approaches compared to the 11-point sequential access P-R curve interpolated in the data set. Comparing the Euclidean and PSNR approaches, it was found that the PSNR method, with an AP value of 0.76, was more sensitive regarding image retrieval.

Performance comparison for all models is given in Table 1.

When Table 1 is examined, the proposed model produces successful results in all four types more than the other compared methods.

## 4. Discussion

Artificial intelligence technology has provided a significant benefit in predictive analyses, medical imaging, pre-operative planning, and post-operative care for various musculoskeletal disorders [26]. A specific area of these focuses on shoulder pathologies, such as rotator cuff tears (RCTs), impingement syndrome, instability, osteoarthritis, and adhesive capsulitis, which have grown rapidly in recent years [27].

AI technology has been used in predictive models to evaluate functional and anatomical outcomes based on a variety of criteria prior to surgery, in addition to diagnosing RCTs. By quantitatively evaluating the fullness of the supraspinatus muscle and utilizing the Otsu thresholding technique on MRI scans to determine the degree of fat infiltration of the supraspinatus muscle, Ro et al. employed a DL model to segment the muscle and fossa areas [28]. A CNN model was created by Taghizadeh et al. to automatically measure and describe rotator cuff muscle degeneration in shoulder CT scans from glenohumeral osteoarthritis patients [29]. By gathering patient data before and after surgery, Potty et al. developed a machine learning (ML) model to forecast post-operative functional results after arthroscopic rotator cuff repair [30]. With AUCs more than 0.99 on internal test sets and greater than 0.97 on external test sets, Wei et al. created CNN models that demonstrated good accuracy in detecting joint dislocations based on radiographs of 140 shoulders and 106 elbows, half of which were dislocated [31]. With 96% accuracy and a 1.00 AUC, a CNN model trained by Chung et al. demonstrated exceptional performance in differentiating between normal shoulder radiographs and proximal humeral fractures [32]. Yu et al. investigated the effectiveness of musculoskeletal ultrasound imaging in conjunction with an intelligent cluster analysis technique for the differential diagnosis and rehabilitation of scapulohumeral periarthritis [33]. In order to categorize inflammation in ultrasound imaging into groups based on its intensity, Lin et al. created an autonomous BPE detection system [34]. Grauhan et al. used 2700 plain radiographs tagged for a few common features to train a CNN model to identify the most prevalent causes of acute or chronic shoulder discomfort. With an AUC of 0.896 for joint dislocation, 0.945 for osteoarthritis, and 0.800 for periarticular calcifications, the CNN model that was built demonstrated great accuracy [35].

No similar study has been found in classification and CBIR regarding detecting acromion types in the examination of shoulder magnetic resonance images. We believe that this is the first time we have addressed this subject. Therefore, a detailed literature review on the subject could not be conducted. However, the biomedical field frequently uses CBIR systems and classification techniques. Since our study will be a pioneering study in the field of CBIR, several different biomedical studies in which CBIR was applied were examined. In one study, Yildirim [36] used the classification and CBIR system to detect bladder cancer. The study’s results obtained using different feature extraction architectures were classified into different classifiers. In addition, the obtained features were evaluated using different similarity measurement metrics. In the study, it was concluded that bladder cancer can be evaluated with CBIR systems. In their study, Sudhish et al. [37] used fuzzy clustering and deep learning techniques to detect brain tumors. The proposed system achieved a success rate of 98.15%, and the researchers stated that the results confirmed its clinical application. Gassner et al. [38] developed a CBIR-based system and used this system for diagnoses in dermatology consultation. They used CNN-based methods in the study. As a result, they stated that this system could increase the diagnostic accuracy and confidence of dermatologists and assistants. They also stated that interpretable methods were presented in the study and that they expected these methods to be more accepted and used in consultation.

The shape of the scapula’s acromion may be related to a number of shoulder conditions, including RCT [2]. Hooked- and curved-type acromions may contribute to tendon traction injury and cause RCT [5]. The reduced size of the subacromial space due to the hooked acromion may also cause more frequent impingement of the rotator cuff tendon and subsequent tearing [9]. Considering such situations, reporting of routine MRI examinations by radiologists taking into account the shape of the acromion would be effective for the clinician to evaluate shoulder pathologies such as RCT or rotator cuff impingement, to have an idea about the etiology and even to determine the treatment protocol.

Although this study is one of the first to determine acromion types in examining shoulder magnetic resonance images, it also has some limitations. The small number of images in the data set is one of this retrospective study’s most significant shortcomings. Secondly, our study is a single-center study. Another limitation may be that it was evaluated based on images from a single sequence of routine MRI examinations. Our future goal is to conduct a more comprehensive study on acromion morphology and its relationship with surrounding structures using MRI images obtained with multiple sequences from different centers.

## 5. Conclusions

In this study, a hybrid CBIR model was developed to detect shoulder acromion types. The study shows that CNN-based deep feature extraction combined with texture-based methods followed by effective feature selection and dimensionality reduction can significantly improve the performance of CBIR systems in identifying acromion shapes in MRI images. The proposed model is promising for accurately and rapidly determining morphological types of the acromion, thus aiding in the diagnosis and understanding of chronic shoulder disorders. PSNR, as a similarity measurement method, has proven to be particularly effective in this context. This study has shown that CBIR-based methods can be used in disease detection in the field of musculoskeletal radiology, and with the determination of acromion shape types in the current study, an awareness of shoulder pathologies and a resource database for future studies have been provided.

## Figures and Tables

**Table 1:** Performance comparison.

Model	Euclidean				PSNR			
	Type 1	Type 2	Type 3	Type 4	Type 1	Type 2	Type 3	Type 4
Densenet201	0.41	0.68	0.32	0.37	0.41	0.68	0.32	0.37
Darknet53	0.43	0.66	0.33	0.38	0.43	0.66	0.33	0.38
Efficientnetb0	0.41	0.73	0.37	0.39	0.41	0.73	0.37	0.39
NasnetMobile	0.40	0.69	0.27	0.30	0.40	0.69	0.27	0.30
LBP	0.40	0.70	0.36	0.44	0.60	0.76	0.63	0.65
HOG	0.40	0.71	0.35	0.49	0.60	0.77	0.59	0.67
Proposed Model	0.58	0.82	0.44	0.64	0.69	0.85	0.63	0.73

## Data Availability

The data set will be shared upon the researchers’ request.

## References

[1] Getz J.D., Recht M.P., Piraino D.W., Schils J.P., Latimer B.M., Jellema L.M., A Obuchowski N. (1996). Acromial morphology: Relation to sex, age, symmetry, and subacromial enthesophytes. Radiology.

[2] Mansur D.I., Khanal K., Haque M.K., Sharma K. (2012). Morphometry of acromion process of human scapulae and its clinical importance amongst Nepalese population. Kathmandu Univ. Med. J..

[3] Khan Y., Nagy M.T., Malal J., Waseem M. (2013). The painful shoulder: Shoulder impingement syndrome. Open Orthop. J..

[4] Oh J.H., Kim J.Y., Lee H.K., Choi J.-A. (2010). Classification and clinical significance of acromial spur in rotator cuff tear: Heel-type spur and rotator cuff tear. Clin. Orthop. Relat. Res..

[5] Bigliani L.U., Ticker J.B., Flatow E.L., Soslowsky L.J., Mow V.C. (1991). Relationship of acromial architecture and diseases of the rotator cuff. Der Orthopade.

[6] Stehle J., Moore S.M., Alaseirlis D.A., Debski R.E., McMahon P.J. (2015). A reliable method for classifying acromial shape. Int. Biomech..

[7] Balke M., Schmidt C., Dedy N., Banerjee M., Bouillon B., Liem D. (2013). Correlation of acromial morphology with impingement syndrome and rotator cuff tears. Acta Orthop..

[8] Gagey N., Ravaud E., Lassau J.P. (1993). Anatomy of the acromial arch: Correlation of anatomy and magnetic resonance imaging. Surg. Radiol. Anat..

[9] Paraskevas G., Tzaveas A., Papaziogas B., Kitsoulis P., Natsis K., Spanidou S. (2008). Morphological parameters of the acromion. Folia Morphol..

[10] Mohamed R.E., Abo-Sheisha D.M. (2014). Assessment of acromial morphology in association with rotator cuff tear using magnetic resonance imaging. Egypt. J. Radiol. Nucl. Med..

[11] Nho S.J., Yadav H., Shindle M.K., MacGillivray J.D. (2008). Rotator cuff degeneration: Etiology and pathogenesis. Am. J. Sports Med..

[12] Morag Y., Jacobson J.A., Miller B., De Maeseneer M., Girish G., Jamadar D. (2006). MR imaging of rotator cuff injury: What the clinician needs to know. Radiographics.

[13] Kashif M., Raja G., Shaukat F. (2020). An efficient content-based image retrieval system for the diagnosis of lung diseases. J. Digit. Imaging.

[14] Phan T.-C., Phan A.-C., Cao H.-P., Trieu T.-N. (2022). Content-based video big data retrieval with extensive features and deep learning. Appl. Sci..

[15] LeCun Y., Bengio Y., Hinton G. (2015). Deep learning. Nature.

[16] Blum A.L., Langley P. (1997). Selection of relevant features and examples in machine learning. Artif. Intell..

[17] Dalal N., Triggs B. Histograms of oriented gradients for human detection. Proceedings of the 2005 IEEE Computer Society Conference on Computer Vision and Pattern Recognition (CVPR’05).

[18] Ding C., Peng H. (2005). Minimum redundancy feature selection from microarray gene expression data. J. Bioinform. Comput. Biol..

[19] İncetaş M.O., Tanyeri U., Kılıçaslan M., Yakışır G.B., Demirci R. Esik Seciminin Benzerlige Dayali Kenar Belirlemeye Etkisi. Proceedings of the Uluslararası Multidisipliner Calismalar ve Yenilikci Teknolojiler Sempozyumu Ismsit 2017.

[20] Smeulders A.W.M., Worring M., Santini S., Gupta A., Jain R. (2000). Content-based image retrieval at the end of the early years. IEEE Trans. Pattern Anal. Mach. Intell..

[21] Smith J.R., Chang S.-F. Tools and techniques for color image retrieval. Proceedings of the Storage and Retrieval for Still Image and Video Databases IV.

[22] Suhasini P.S., Krishna K., Krishna I.V.M. (2009). CBIR using color histogram processing. J. Theor. Appl. Inf. Technol..

[23] Information Retrieval: How Search Engines Retrieve Data—IONOS. https://www.ionos.co.uk/digitalguide/online-marketing/search-engine-marketing/information-retrieval-how-search-engines-retrieve-data/.

[24] Schütze H., Manning C.D., Raghavan P. (2008). Introduction to Information Retrieval.

[25] Ioannakis G., Koutsoudis A., Pratikakis I., Chamzas C. (2017). Retrieval an online performance evaluation tool for information retrieval methods. IEEE Trans. Multimed..

[26] Cheng C., Liang X., Guo D., Xie D. (2024). Application of Artificial Intelligence in Shoulder Pathology. Diagnostics.

[27] Familiari F., Galasso O., Massazza F., Mercurio M., Fox H., Srikumaran U., Gasparini G. (2022). Artificial Intelligence in the Management of Rotator Cuff Tears. Int. J. Environ. Res. Public Health.

[28] Ro K., Kim J.Y., Park H., Cho B.H., Kim I.Y., Shim S.B., Choi I.Y., Yoo J.C. (2021). Deep-learning Framework and Computer Assisted Fatty Infiltration Analysis for the Supraspinatus Muscle in MRI. Sci. Rep..

[29] Taghizadeh E., Truffer O., Becce F., Eminian S., Gidoin S., Terrier A., Farron A., Büchler P. (2021). Deep Learning for the Rapid Automatic Quantification and Characterization of Rotator Cuff Muscle Degeneration from Shoulder CT Datasets. Eur. Radiol..

[30] Potty A.G., Potty A.S.R., Maffulli N., Blumenschein L.A., Ganta D., Mistovich R.J., Fuentes M., Denard P.J., Sethi P.M., Shah A.A. (2023). Approaching Artificial Intelligence in Orthopaedics: Predictive Analytics and Machine Learning to Prognosticate Arthroscopic Rotator Cuff Surgical Outcomes. J. Clin. Med..

[31] Wei J., Li D., Sing D.C., Beeram I., Puvanesarajah V., Tornetta P., Fritz J., Yi P.H. (2022). Detecting Upper Extremity Native Joint Dislocations Using Deep Learning: A Multicenter Study. Clin. Imaging.

[32] Chung S.W., Han S.S., Lee J.W., Oh K.S., Kim N.R., Yoon J.P., Kim J.Y., Moon S.H., Kwon J., Lee H.J. (2018). Automated Detection and Classification of the Proximal Humerus Fracture by Using Deep Learning Algorithm. Acta Orthop..

[33] Yu L., Li Y., Wang X.F., Zhang Z.Q. (2023). Analysis of the Value of Artificial Intelligence Combined with Musculoskeletal Ultrasound in the Differential Diagnosis of Pain Rehabilitation of Scapulohumeral Periarthritis. Medicine.

[34] Lin B.S., Chen J.L., Tu Y.H., Shih Y.X., Lin Y.C., Chi W.L., Wu Y.C. (2020). Using Deep Learning in Ultrasound Imaging of Bicipital Peritendinous Effusion to Grade Inflammation Severity. IEEE J. Biomed. Health Inform..

[35] Grauhan N.F., Niehues S.M., Gaudin R.A., Keller S., Vahldiek J.L., Adams L.C., Bressem K.K. (2022). Deep Learning for Accurately Recognizing Common Causes of Shoulder Pain on Radiographs. Skelet. Radiol..

[36] Yildirim M. (2024). Content-Based Image Retrieval and Image Classification System for Early Prediction of Bladder Cancer. Diagnostics.

[37] Sudhish D.K., Nair L.R., Shailesh S. (2024). Content-based image retrieval for medical diagnosis using fuzzy clustering and deep learning. Biomed. Signal Process. Control.

[38] Gassner M., Garcia J.B., Tanadini-Lang S., Bertoldo F., Fröhlich F., Guckenberger M.,  Braun R.P. (2023). Saliency-enhanced content-based image retrieval for diagnosis support in dermatology consultation: Reader study. JMIR Dermatol..

